# The blind men and the elephant: The case for a transdiagnostic approach to initiation

**DOI:** 10.3389/fpsyg.2022.1113579

**Published:** 2023-02-07

**Authors:** Karen Leneh Buckle, Ellen Poliakoff, Emma Gowen

**Affiliations:** Body, Eye and Movement Lab, Division of Psychology, Communication and Human Neuroscience, Faculty of Biology, Medicine and Health, University of Manchester, Manchester, United Kingdom

**Keywords:** initiation, autism, schizophrenia, Parkinson’s, catatonia, transdiagnostic, motivation, movement

## Abstract

Difficulty initiating voluntary action is an under-recognized and often invisible impairment in various psychiatric, neurodevelopmental, and neurological conditions. Understanding the commonalities of volition impairments across diagnoses is limited by a lack of consistent terminology, arbitrary distinctions between conditions, the habit of looking only to the prevailing definitions and theories to explain observed traits, and the covert nature of initiation. The siloed approach to research in this area evokes the parable of the blind men and the elephant, where understanding the whole picture is impeded by a limited view. There has been little effort to consider how differing terms overlap or to use objective methods to differentiate phenomena along meaningful lines. We propose a triad of interacting elements, all of which are needed for successful initiation of voluntary action: (i) executive function, (ii) volition, and (iii) movement. Failure to initiate a response may be due to impairments in any of these, which often co-occur. This paper calls for the following considerations to improve research in this area: (i) put aside preconceptions about conditions and their mechanisms to adopt a flexible transdiagnostic approach; (ii) consider executive function, movement, and volition as possible dimensional variations with related underlying mechanisms; (iii) carefully differentiate components of complex functions; (iv) look to first-hand reports for covert and previously unrecognized traits. These approaches have the potential to elucidate the cognitive and biological mechanisms underpinning voluntary action and create a foundation to develop more appropriate and informed interventions.

## 1. Introduction

Difficulty initiating voluntary action occurs in various psychiatric, neurodevelopmental, and neurological conditions, but is not often recognized as a distinctive or important characteristic. Research on this topic has been inhibited by a siloed approach that evokes the parable of the blind men and the elephant. In this story, when six blind men encounter an elephant, each describes a very different impression (Saxe, 1884). Just as the blind men describe the elephant as like a snake, tree or fan depending on whether they are touching the trunk, a leg, or an ear, so our present-day health care providers, educators and researchers describe impairments in initiation in terms of their perspective. In this way, a neurologist sees initiation impairments in Parkinson’s disease, attributes them to a neurological deficit, and calls them akinesia or apathy (Radakovic and Abrahams, 2018); a psychiatrist sees a lack of goal directed activity in schizophrenia, considers it a lack of motivation, and calls it avolition (e.g., Mucci et al., 2015); a behavior therapist sees an autistic child’s failure to respond to an instruction, considers it behavioral, and does not acknowledge it as a separate issue at all (e.g., Koegel and Mentis, 1985; Chevallier et al., 2012). Understanding the commonalities of volition impairments across diagnoses is impeded by a lack of consistent terminology, a tendency to pigeonhole conditions into categories such as ‘neurological’ and ‘psychiatric’, defining conditions solely based on overt characteristics, and the habit of looking only to the prevailing definitions and theories to explain observed traits.

While there is evidence of initiation impairments across many neurological, neurodevelopmental and psychiatric conditions, this paper will refer mainly to three example conditions to demonstrate the arguments: Parkinson’s disease, schizophrenia, and autism. Initiation impairments are evident in these three conditions, yet similarities and possible underlying mechanisms are currently underexplored. The exploration in this paper will use autism as a focal point due to the expertise of the authors and the under-recognition of initiation impairments as a feature of autism in particular.

## 2. Barriers to recognition of initiation impairments

### 2.1. Disjointed terminology

The lack of communication across disciplines and specialties leads to similar phenomena being described in different terms and explained with different theory based on limited formal definitions. In a preliminary review of the literature, roughly 30 terms have been found for a range of poorly defined symptoms or syndromes characterized by deficits in initiating action (Table 1) in clinical and non-clinical populations.

**Table 1:** Terms for initiation impairments across clinical and non-clinical groups.

Term	Meaning or usage	Common contexts	Example(s)
Abulia	Loss of will and drive.	Acquired brain injury	Yamanaka et al. (1996)
Adynamia	Difficulty initiating and completing tasks.	Acquired brain injury	Queensland Health (2017)
Akinesia	Staying still for extended periods or having severe difficulty initiating movements. Also ‘akinetic mutism’.	Catatonia	Northoff et al. (1995)
Amotivation	An umbrella term for initiative-related symptoms in psychiatric conditions.	Schizophrenia Cannabis abuse	Foussias and Remington (2010)
Analysis paralysis	Tendency to get stuck in analyzing all possible outcomes to the point that it interferes with decision making.	General population Obsessive Compulsive Disorder	Zuckerberg and Gibbs (2008)
Apathy	Lack of emotion-driven activity, or lack of caring about what happens.	Parkinson’s disease Schizophrenia Dementia	Foussias and Remington (2010)
Athymhormia	A profound lack of self-motivation which may be so severe as to be life threatening.	Basal ganglia dysfunction (by disease or injury)	Habib (2004)
Auto-activation deficit	An absence of internal trigger to move, with intact response to external prompts.	Basal ganglia injury	Laplane and Dubois (1998)
Attenuated behavior	Catatonia-like characteristics including slow movement, freezing and prompt dependence	Neurodevelopmental and genetic syndromes	Breen (2014)
Avoidance	Using other tasks or excuses to avoid something unpleasant, difficult, or frightening.	Anxiety Avoidant Personality Disorder	Schmidt et al. (2008)
Avolition	Lack of will, drive and spontaneity, especially as a symptom.	Schizophrenia	Mucci et al. (2015)
Catatonia	A motor syndrome characterized by difficulty initiating and completing movements.	Schizophrenia Autism	Bell et al. (2019)
Cognitive paralysis	The tendency to freeze due to an inability to reach and act on a decision, especially in an emergency situation.	General population	Leach (2005)
Demand avoidance	Actively refusing to respond to requests or demands of others.	Autism ADHD	Gillberg et al. (2015)
Executive dysfunction	Impairments in the executive system which interrupts automatic responses and allows an adaptive response to be selected.	ADHD Autism	Bramham et al. (2009)
Freezing	A response to threat, as in ‘fight, flight, freeze’.	General population Anxiety disorders	Epstein and Silbersweig (2015)
Hypokinesia	Lack of movement. Usually, a delay in initiating movement responses.	Parkinson’s disease	Schilder et al. (2017)
Initiation impairment	Difficulty initiating and completing tasks.	Acquired brain injury	Queensland Health (2017)
Laziness	Being unwilling to work.	General population	
Learned helplessness	A state of diminished motivation brought about by actions persistently failing to produce desired effect.	Any condition where behavior intervention is used.	Koegel and Mentis (1985)
Non-compliance	Not doing what someone in authority requests or requires.	Autism Most psychiatric conditions	Ming et al. (2004)
Oppositional defiance	Actively refusing to respond to requests or demands of others.	Autism ADHD	Gadow et al. (2008)
Obsessional slowness	Slow movements combined with repetitive and/or obsessional behavior	Obsessive Compulsive Disorder (OCD)	Ganos et al. (2015)
Prompt dependence	A state of diminished motivation in which the person can only act when prompted.	Any condition where behavior intervention is used.	Scattone and Knight (2006)
Procrastination	The voluntary delaying of an undesirable task.	General population	
Stupor	Non-responsiveness to the environment	Catatonia	Wilson et al. (2015)
Unmotivated	Not having interest or enthusiasm, especially for social interaction	General population	

In the mid-20th century, the term *athymhormia,* derived from the Greek for ‘no emotion drive’, was coined for the lack of internal drive in people with schizophrenia (Habib, 2004). This term has been largely replaced by *avolition* (Foussias and Remington, 2010), but is still occasionally used for the loss of drive in basal ganglia dysfunction, which itself is more often called *auto-activation deficit* (Laplane and Dubois, 2001). A rarely diagnosed syndrome called *obsessional slowness* is characterized by “profound difficulties in initiating voluntary action, slowness of movement, poor speech production, motor perseverations, and abnormal posturing” (Ganos et al., 2015). This description is identical to *catatonia,* which at its most extreme may be manifest as minimal response to the environment (*stupor*).

While impaired motivation is seen as fundamental to psychotic conditions, similar difficulties are rarely identified in neurodevelopmental conditions such as autism and attention deficit hyperactivity disorder (ADHD). A lack of goal directed activity or response to instructions in these conditions is typically labelled with terms such as *oppositional defiance*, *non-compliance*, and *demand avoidance* (Gadow et al., 2008). In opposition to this behavioral perspective, autistic people have developed their own terminology around *inertia* (Buckle et al., 2021) and *monotropism* (Murray et al., 2005) to describe a common but under-recognized tendency to become ‘stuck’, unable to initiate or change activity in spite of motivation to do so. In brain injury and degenerative neurological disorders, a lack of action is more likely to be described with movement-related terminology such as *akinesia*, *adynamia* and *hypokinesia* (Heilman, 2011). When the lack of activity is also associated with a lack of emotional drive, it is often called *apathy*.

The distinction between these terms is, at best, subtle, and there has been little effort to consider how differing terms overlap or to use objective methods to differentiate phenomena along meaningful lines. Instead, each field of research approaches the symptoms as if they are unique to their condition of interest, attempts to explain it in terms of the identified ‘core characteristics’ of that condition, and gives it a new name. To avoid this problem, this paper’s authors opted to use a term that is both inclusive and descriptive without presupposing a specific underlying mechanism: *initiation impairments.*

### 2.2. Conceptual pigeonholing

Longstanding yet arbitrary distinctions between neurological, psychiatric, and neurodevelopmental disorders affects the way initiation impairments are constructed and explained. For example, autism has been defined primarily in terms of social impairments and repetitive behavior. Despite the extreme heterogeneity in autistic presentation, research tends to frame all autistic differences in terms of these ‘core characteristics’. So, when an autistic person does not respond to a request, it is seen as willful refusal to cooperate. Similarly, a lack of social initiation is seen as the outcome of a lack of social motivation (Koegel and Mentis, 1985; Chevallier et al., 2012). These assumptions can lead to inappropriate behavioral interventions, as in the case report by Ming et al. (2004) where an autistic woman who had been subjected to behavior modification interventions for ‘non-compliance’ was shown to be exerting measurable mental effort that was not manifest in physical action. Some researchers have recognized the importance of autistic differences in sensory, motor (Donnellan et al., 2013), executive (Ozonoff et al., 1991), or arousal regulation (Welch et al., 2020). These approaches would support the idea that such processing differences may underlie such characteristics as non-compliance, passivity, and withdrawal; however, they remain alternative rather than mainstream views.

In schizophrenia, alongside the positive psychotic symptoms, there is a cluster of negative symptoms characterized by withdrawal and lack of motivation (Lim et al., 2016). As with autism, these more mundane symptoms may be overshadowed by the more prominent symptoms of delusions and hallucinations. In Parkinson’s disease, the inverse is true. Difficulty initiating movement is fundamental to Parkinson’s, and freezing in place is a highly visible manifestation. Because of this, in Parkinson’s, difficulty with initiating and sustaining action is more readily recognized than in conditions where the initiation difficulties lack such overt expression; however, non-motor symptoms of Parkinson’s such as sleep disturbance, apathy, and executive dysfunction are relatively neglected (Todorova et al., 2014).

It is evident from this brief survey of syndromes and symptoms that the importance of characteristics outside those considered core characteristics of a condition are often overlooked. Recent trends towards valuing and exploring the lived experience of neurological and psychiatric conditions can draw attention to covert traits and their impact (e.g., Carlsson et al., 2004; Hartley et al., 2014; Butcher et al., 2020; Buckle et al., 2021) and offer alternative explanations to prevailing theories (Kapp et al., 2019). There is increasing pressure from autistic people for researchers and clinicians to recognize that observable autistic behavior is the outcome of a differently functioning brain (Woods et al., 2018). For example, the significant difficulties that autistic people have with acting at will have only just begun to be recognized in community-driven research (Buckle et al., 2021).

Because of the disparate terminology, conceptual separation of neurological and psychiatric, and the bias towards those characteristics that are included in formal clinical descriptions, the theoretical underpinnings of initiation impairments are similarly incoherent. A key to improving research in this area is to start looking beyond these arbitrary boundaries and adopt a coherent and flexible transdiagnostic approach.

## 3. Transdiagnostic approach

A few researchers have taken a transdiagnostic approach to understanding symptoms of neurological and psychiatric conditions more broadly. Negative symptoms usually associated with schizophrenia have been investigated across multiple psychiatric and neurological conditions. Strauss and Cohen’s (2017) analysis notes several negative symptoms such as alogia and asociality (lack of speech or interaction, respectively) in autistic people; however, they did not identify avolition as an autistic feature because this is not yet widely recognized in autism. Another study comparing only autism and schizophrenia found considerable overlap in characteristics such as passivity, diminished social initiation, and reduced speech production (Trevisan et al., 2020). Autism, Parkinson’s disease, and schizophrenia overlap substantially in their deficit or ‘negative’ symptoms, and the ability to formulate and act on an intention is central to these deficits.

Catatonia is a transdiagnostic psychomotor syndrome occurring at greatly elevated rates in psychiatric and neurological conditions, including autism (Wing and Shah, 2000). Difficulty initiating and completing movements and prompt dependence are core features, with additional symptoms including repetitive behavior, difficulty with decision making and unresponsiveness. Motor traits include Parkinsonian traits such as rigidity, slowness, freezing, and gait alterations. Catatonia is usually only recognized in a severe form, but milder expressions characterized by passivity, prompt dependence, and lack of initiative (Wing and Shah, 2006) share many characteristics with apathy and the negative symptoms of schizophrenia, avolition in particular.

### 3.1. Initiation triad

A further difficulty understanding initiation impairments is that initiation is an outcome of multiple cognitive functions with unclear boundaries. There is little agreement on where a neurogenic inability to initiate ends and a psychogenic lack of motivation begins. Similarly, it is unclear how, or if, difficulty executing an action may arise from executive function planning deficits versus a motor initiation problem such as that seen in Parkinson’s.

**Figure 1:**
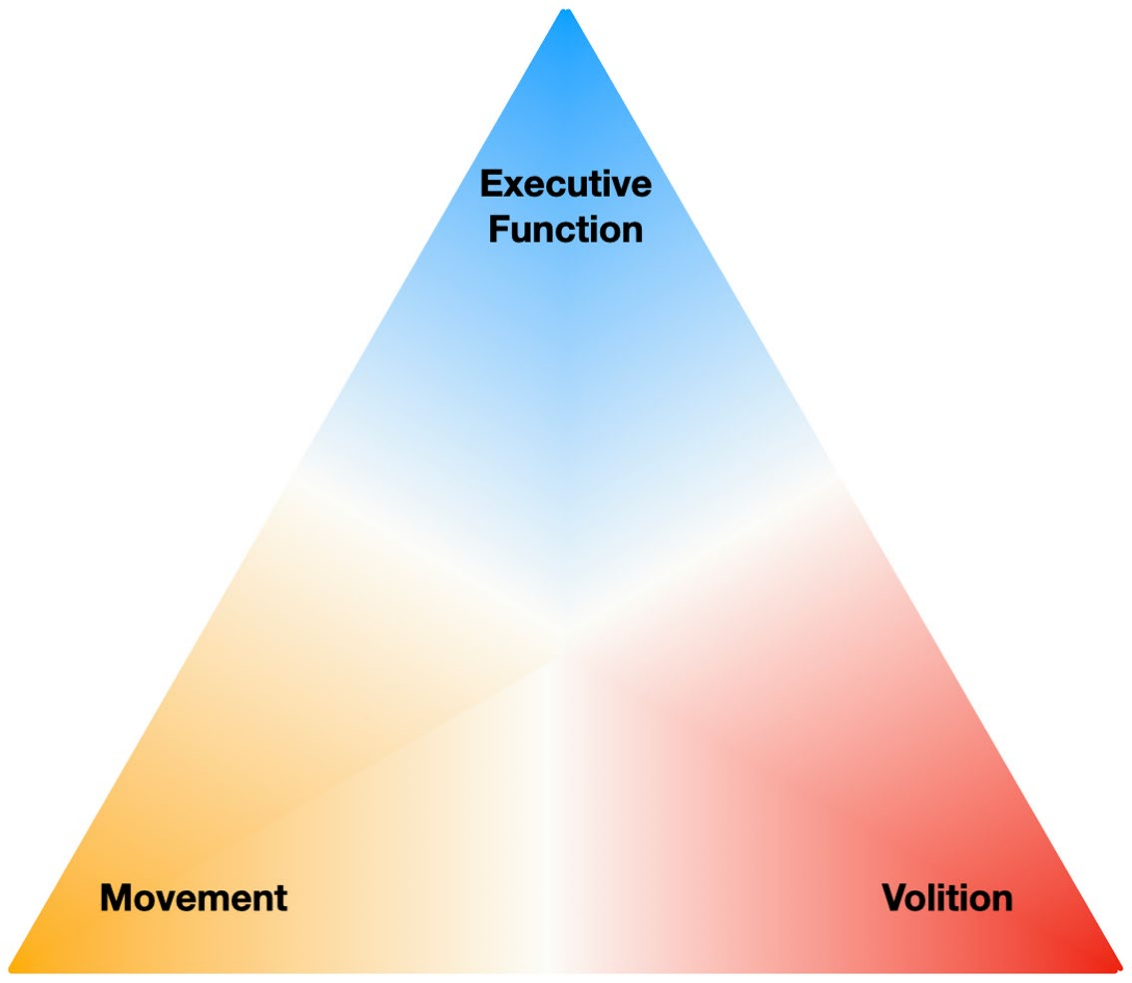
The three interacting elements of an ‘initiation triad’.

As an alternative to attempting to clearly distinguish these blurred dimensions, initiation may be viewed in terms of interacting elements, all of which are needed for successful initiation of goal-directed behavior. The execution of an action requires high level processes such as planning and decision making, the emotional drive or motivation to act, and the physical ability to move. Thus, we propose a triad of contributors to initiation of voluntary action: (i) executive function, (ii) volition (or motivation), and (iii) movement (Figure 1). Failures to initiate action are often explained in terms of just one of these, as indicated by the theoretical constructs of a specific condition; however, the boundaries between these elements are blurred and impairments in all three often co-occur.

#### 3.1.1. Executive function

While research in executive functions typically focus on planning, inhibition, and working memory, initiation is also an aspect of executive function related to cognitive flexibility and the selection of a desired response (Banich, 2009; Bramham et al., 2009). Executive function has been extensively studied in autism (Demetriou et al., 2018), but only one study known to these authors has looked specifically at initiation of a response (Carmo et al., 2017). This study found that autistic people were impaired only in the initial interval of a verbal fluency task, and that this difference could be eliminated by providing a starting point. These findings provide strong evidence for an initiation-specific impairment. Executive deficits are also well documented in neurological conditions such as Parkinson’s (Kudlicka et al., 2011) and a wide variety of psychiatric conditions (Banich, 2009).

#### 3.1.2. Volition

The volition element is difficult to capture and may be variously conceptualized as motivation, arousal, or drive. Motivational deficits are most prominent in psychiatric conditions such as depression and schizophrenia, where they can be so pronounced that the person is unable to care for their basic needs (Butcher et al., 2020). Autistic people are also frequently very passive, which may be a risk factor for catatonia (Shah, 2019). When autistic people talked about their difficulty doing things, a sense of being ‘switched off’ was noted, including a lack of distress about the inability to move (Buckle et al., 2021). Apathy, characterized by lack of motivation and drive, is increasingly recognized and researched in Parkinson’s (Todorova et al., 2014). Impairments in drive may also be related to difficulty with arousal regulation, as someone who is under-aroused will have a higher threshold for any action. Difficulties with arousal regulation are manifest in autistic response to stress, including phenomena commonly called shutdown and meltdown (Shah, 2019; Phung et al., 2021).

#### 3.1.3. Movement

As with executive function, movement is a broad and complex area of which initiation is one part. Difficulty initiating movement is a hallmark of Parkinson’s disease, even more fundamental than the characteristic tremor. Motor differences have also been noted in autism since its first descriptions, yet significant motor symptoms are usually considered peripheral or additional to autism (Donnellan et al., 2013). As many as 80% of autistic people have difficulty with motor coordination (Gowen and Hamilton, 2013) and up to 40% have significant features of catatonia (Breen and Hare, 2017). Impairment in initiation of voluntary movements has been described by autistic people themselves (Welch et al., 2020; Buckle et al., 2021), and older autistic adults report elevated rates of Parkinsonian features (Geurts et al., 2022).

The co-occurrence of the three elements of the initiation triad, as well as the unclear boundaries between them suggest a related underlying mechanism. Evidence for a relationship between motor and executive functions has been found in degenerative conditions such as Parkinson’s as well as in normal and atypical childhood development such as premature birth and developmental coordination disorder (dyspraxia; Van der Fels et al., 2015; Bernardi et al., 2018). Outside of clinical populations, there is a strong developmental relationship between executive function and motor control, including the development of hand preference (Gonzalez et al., 2014). The third element, volition impairment, is less often included in studies that involve initiation, but such traits can be observed alongside psychomotor slowing and executive dysfunction in depression (Snyder, 2013) and dementia (Boyle et al., 2003).

A coherent approach to initiation impairments requires simultaneously recognizing the similarities in symptoms across conditions and differentiation of components of cognitive domains into meaningful components. Behavior-based clinical descriptions have traditionally included five negative symptoms of schizophrenia, but more recent studies have shown these can be more meaningfully grouped as two clusters. The volitional cluster includes apathy, avolition, and lack of motivation, whereas the expressive cluster includes characteristics such as anhedonia, flattened affect, and asociality (Lim et al., 2016). In a study of the relationship between psychomotor and negative symptoms, Bervoets et al. (2014) found that deficits in volition, but not expression, were associated with difficulty initiating movements. This was an initiation-specific association that was not duplicated in planning or execution of movements once started. Conventional approaches which fail to distinguish the components of negative symptoms, apathy, or executive functions may obscure such associations with specific dimensions of these complex functions.

### 3.2. Implications

Recognizing the contribution of cognitive, emotional, and movement elements to initiation, and the presence of all of these in neurodevelopmental, neurodegenerative, and psychiatric conditions, can elucidate the cognitive and biological mechanisms underpinning voluntary action and create a foundation to develop more appropriate and informed interventions. The understanding of the neurology of autism is still in its infancy and is impeded by the heterogeneity of the condition as well as the diffuse symptoms. Careful differentiation of components of complex functions along with cluster analysis and similar techniques may help to clarify these relationships.

The ability to theorize about intention, motivation, and initiation based only on observation is necessarily limited due to the covert nature of action initiation. While it is now recognized that autism is a neurological condition, there is still a tendency to view autistic behavior as social, emotional and volitional. In recent qualitative research, autistic people have explained that in contrast to conventional explanations based on deficient social motivation, even when they are highly motivated by serious consequences or physical discomfort, they are still unable to act (Buckle et al., 2021). A few researchers have proposed rethinking many autistic traits that are normally considered social–emotional and viewing them instead as manifestations of sensory and motor differences (Donnellan et al., 2013; Torres, 2015). Such ‘autistic behaviors’ as non-compliance, lack of communication, lack of affect, and resistance to change could be due to difficulties with initiation of a response (Welch et al., 2018). Recognizing the similarities between autistic initiation impairments and those of people with specific neurological conditions may indicate candidate brain structures for investigation in autism.

Understanding the various contributors to a lack of initiation and recognizing the similarities of initiation impairments across conditions has the potential to support more appropriate interventions. Autistic people are clear that they are often unable, not unwilling, to act, but the assumption that non-compliance is a choice can lead to punishment or inappropriate interventions (Ming et al., 2004). If someone with Parkinson’s fails to respond to an instruction, it is clear that this is due to their motor impairment and that rewards are unlikely to be helpful, yet when an autistic person similarly fails to act, behavioral approaches are the standard. If motivation is not the problem, then reward is not the solution. Acknowledging the possibility of a movement element of failure to act in people with schizophrenia and autism may open the door to trialing movement-oriented interventions shown to help people with Parkinson’s overcome freezing.

## 4. Conclusion

It has long been recognized that the distinction between neurological and psychiatric conditions is arbitrary, yet circumscribed understandings based on ‘core features’ and social–emotional explanations continue to limit research and practice in neuropsychiatric conditions. Auto-activation deficits and apathy in neurological disorders, executive function impairments in neurodevelopmental conditions, psychomotor slowing and amotivation/avolition in psychiatric conditions are all interrelated. Putting aside preconceptions about conditions allows us to see commonalities such as the co-occurrence of executive, motor and volitional impairments. Beyond that, differentiating the components of complex constructs such as ‘executive function’ and ‘apathy’ allows relationships between volition, motor and executive to be seen. Failure to initiate a response may be due to impairments in executive function, motor control, or an outcome of a lack of drive. Research approaching these apparently separate functions as possibly dimensional variations with a related underlying mechanism may improve understanding of motivation and initiative more broadly. Most importantly, recognizing the difference between a social-emotionally motivated refusal to comply and a neurological inability to initiate a response can avoid inappropriate and even inhumane treatment.

## Data availability statement

The original contributions presented in the study are included in the article/supplementary material, further inquiries can be directed to the corresponding author.

## Author contributions

KB initiated the ideas in the paper and wrote the first draft of the manuscript. EP and EG contributed to the development of ideas and reviewed drafts of the article. All authors contributed to the article and approved the submitted version.

## Funding

This research forms part of a PhD funded by the John and Lorna Wing foundation.

## Conflict of interest

The authors declare that the research was conducted in the absence of any commercial or financial relationships that could be construed as a potential conflict of interest.

## Publisher’s note

All claims expressed in this article are solely those of the authors and do not necessarily represent those of their affiliated organizations, or those of the publisher, the editors and the reviewers. Any product that may be evaluated in this article, or claim that may be made by its manufacturer, is not guaranteed or endorsed by the publisher.
